# The effects of compassion and fears of compassion on mental health during the COVID-19 pandemic: A multinational study across 21 countries

**DOI:** 10.1192/j.eurpsy.2022.232

**Published:** 2022-09-01

**Authors:** M. Matos

**Affiliations:** University of Coimbra, Center For Research In Neuropsychology And Cognitive Behavioral Intervention (cineicc), Coimbra, Portugal

**Keywords:** Compassion, mental health, Multinational study, Covid-19

## Abstract

**Introduction:**

The COVID-19 pandemic is having an unprecedented detrimental impact on mental health in people around the world. It is therefore important to examine factors that may buffer or heighten the risk of mental health problems in this context.

**Objectives:**

This study explores the buffering effects of different flows of compassion (for self, for others, from others) and the magnifying effects of fears of compassion on the impact of perceived threat of COVID-19 on depression, anxiety and stress, and social safeness.

**Methods:**

4057 adult participants collected from the general community population across 21 countries from Europe, Middle East, North America, South America, Asia and Oceania, completed self-report measures of perceived threat of COVID-19, compassion, fears of compassion, depression, anxiety, stress, and social safeness.

**Results:**

Self-compassion moderated the impact of perceived threat of COVID-19 on depression, anxiety and stress, whereas compassion from others moderated the effects of fears of COVID-19 on social safeness. Fears of compassion moderated the impact of perceived threat of COVID-19 on psychological distress. Only fears of compassion from others moderated the effects of fears of COVID-19 on social safeness. These effects were consistent across countries.

**Conclusions:**

Our findings highlight the universal protective role of compassion, in particular self-compassion and compassion from others, in promoting resilience by buffering against the harmful effects of the COVID-19 pandemic on mental health and social safeness. Furthermore, our results reveal that fears of compassion have a magnifying effect on the damaging impact of the COVID-19 pandemic on mental health and social safeness.

**Disclosure:**

I wasn’t able to add the full list of authors above. Please add the full list as described below.

